# Quercetin Enhances Gastric Nitric Oxide Formation and Potentiates the Antihypertensive Effects of Oral Nitrite Administration

**DOI:** 10.1111/bcpt.70211

**Published:** 2026-02-11

**Authors:** Mila Silva‐Cunha, Sandra O. Conde‐Tella, Macário A. Rebelo, Isadora L. M. Kemmer, Ana K. Lima‐Silva, Ediléia de S. P. Caetano, Jose E. Tanus‐Santos

**Affiliations:** ^1^ Department of Pharmacology, Ribeirao Preto Medical School University of Sao Paulo Ribeirao Preto Brazil; ^2^ Department of Translational Medicine State University of Campinas Campinas Brazil; ^3^ School of Pharmaceutical Sciences of Ribeirao Preto University of Sao Paulo Ribeirao Preto Brazil

**Keywords:** antioxidants, blood pressure, nitric oxide, nitrosylation, sodium nitrite

## Abstract

Oral nitrite supplementation enhances nitric oxide (NO) bioavailability and lowers blood pressure. Quercetin may facilitate the reduction of nitrite to NO. However, the combined effects of nitrite and Quercetin on gastric NO formation and blood pressure have not been explored. We investigated whether oral treatment with Quercetin enhances the gastric conversion of nitrite to NO (in vitro and in vivo) and exerts antioxidant and antihypertensive effects. Spontaneously hypertensive rats (SHR) were divided into six groups (*n* = 6/group): three groups treated with Quercetin 10 mg/kg followed 15 min later by water, nitrite 1 mg/kg (nonantihypertensive) or 15 mg/kg (antihypertensive) by gavage, and three similar control groups treated with vehicle, followed by the same nitrite treatments. Blood pressure, gastric NO, plasma nitrite, nitrate, nitrosylated species (RxNO) and aortic S‐nitrosylated proteins were measured. Oxidative stress was also assessed. Quercetin treatment converted a nonantihypertensive dose of nitrite into an effective antihypertensive intervention in association with increased in vitro and in vivo gastric NO formation. However, Quercetin did not enhance nitrite‐induced increases in systemic or vascular nitrite, nitrate, RxNO concentrations nor vascular protein nitrosation. Our results show that Quercetin is a potent enhancer of gastric NO formation from nitrite and amplifies its antihypertensive effects. This combination of drugs may represent a safe and effective therapeutic strategy.

## Plain Language Summary

Nitric oxide (NO) is important to control blood pressure. Sodium nitrite can increase NO levels, and quercetin, a common dietary antioxidant, may enhance this effect. In this study, hypertensive rats received nitrite with or without quercetin. Quercetin boosted the stomach’s ability to convert nitrite into NO, turning a low, otherwise ineffective nitrite dose into one that lowered blood pressure. This effect was linked to increased NO formation in the stomach, but not in the blood or vessels. Taken together, quercetin and nitrite may safely improve blood pressure control.

## Graphical abstract

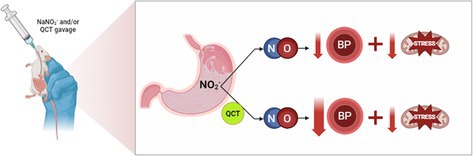

## 1 Introduction

Nitric oxide (NO) is recognized as a crucial gaseous signalling molecule with a pivotal role in cardiovascular homeostasis. Endothelial cells synthesize NO by enzymatically converting L‐arginine through endothelial NO synthase. In the circulation, NO facilitates the relaxation of blood vessel muscles and inhibits platelet activation and immune cell adhesion [1]. Beyond vasodilation, NO and other related nitroxides may form nitrosylated compounds and promote nitrosation reactions by increasing the concentrations of S‐nitrosothiols (RSNO), which serve as significant signalling molecules, influence protein function, gene expression and cellular redox status [2, 3].

In addition to the enzymatic pathway, NO can also be generated from inorganic nitrate (NO_3_^−^) and nitrite (NO_2_^−^) through the entero‐salivary cycle of the NO_3_^−^ [4, 5, 6]. This cycle encompasses the conversion of dietary NO_3_^−^ from NO_3_^−^‐rich foods into NO_2_^−^ by oral bacteria, absorption of NO_2_^−^ into the bloodstream and further conversion into NO by enzymes with nitrite‐reductase activity. Indeed, NO_2_^−^ can is converted into NO via various pathways, including enzymatic NO_2_^−^ reduction facilitated by haemoglobin, myoglobin, xanthine oxidoreductase and mitochondrial enzymes thus resulting in therapeutic effects [4, 5, 7, 8]. In addition, when NO_2_^−^‐enriched saliva reaches the acidic conditions of the stomach, NO_2_^−^ undergoes chemical reactions and generates nitrogen oxides that increase the gastric formation of RSNO and contribute to the cardiovascular effects of nitrate and nitrite [9, 10]. This mechanism has been critically implicated in the cardiovascular responses to nitrite, and any factors increasing gastric pH result in blunted blood pressure responses [11, 12, 13, 14]. Conversely, some antioxidants, such as ascorbic acid and other dietary phenolic compounds [15], enhance NO formation from nitrite by acting as potent reducing agents, potentiating the cardiovascular responses [16, 17].

Quercetin, a flavonoid present in vegetables, exhibits robust antioxidant properties and potential antihypertensive effects. Although unproven, a possible mechanism explaining it effects is the ability to enhance NO production by serving as a reducing agent in the gastric conversion of NO_2_^−^ to NO or by promoting antioxidant effects [18, 19, 20, 21, 22, 23, 24]. In fact, strategies aimed at increasing NO bioavailability while minimizing nitrosative stress hold promise for the treatment of hypertension and other cardiovascular diseases. An understanding of the mechanisms governing these reactions, the influence of gastric pH, and the impact of antioxidants can provide valuable insights for interventions designed to control and prevent such diseases.

Building on these findings, we tested the hypothesis that oral Quercetin treatment could potentiate the established antihypertensive effects of oral sodium nitrite (NaNO_2_) supplementation by enhancing gastric NO production. Moreover, we examined the impact of Quercetin and NaNO_2_ co‐administration on protein nitrosation because this posttranslational modification has been implicated in the antihypertensive responses to oral nitrite administration [25, 26]. Finally, we examined how Quercetin and nitrite treatment may interact to produce antioxidant effects. Examining these mechanisms may improve our understanding of how Quercetin may be very useful in the therapy of hypertension and other cardiovascular diseases.

## 2 Material and Methods

### 2.1 Assessment of In Vitro Effects of Quercetin on NO Formation From NaNO_2_

To investigate in vitro effects of Quercetin on NO formation from NaNO_2_, we employed a simulated gastric juice (SGJ) solution composed of 7 mM HCl and 50 mM NaCl in distilled water, with its pH adjusted to 2.0 by adding HCl [15]. NO production was monitored using 2 mL of SGJ solution containing NaNO_2_ at concentrations of 1, 2.5, 5 or 7.5 μM, in the presence or absence of Quercetin at 10 mM. NO production was detected by chemiluminescence using a NO analyser (Ecomedics Analyzer CLD 88 sp.; Duernten, Switzerland). In essence, we examined whether Quercetin enhances NO formation from NaNO_2_. The maximum chemiluminescence signal (mV) was measured after each NaNO_2_ injection, and the amounts of NO generated from nitrite were calculated.

### 2.2 Animal Model

The study was conducted in accordance with the Basic & Clinical Pharmacology & Toxicology policy for experimental and clinical studies [27]. The present study and experimental procedures complied with the ARRIVE guidelines and were in accordance with the National Research Council's Guide for the Care and Use of Laboratory Animals. The study was approved by the Animal Care and Use Committee of Ribeirao Preto Medical School—University of Sao Paulo (Protocol Code 1748/2023R1). Male Wistar rats (weighing between 200 and 250 g) were obtained from the breeding facility at the Ribeirão Preto campus of the University of Sao Paulo, while male spontaneously hypertensive rats (SHR) were obtained from the Rat Production Facility at the Institute of Biomedical Sciences, University of Sao Paulo. The animals were 10 weeks old and were housed in the maintenance facility with unrestricted access to food and water, in rooms with a 12‐h light/dark cycle, at a controlled temperature (22°C–25°C).

### 2.3 Drugs and Treatment

The animals were divided into six treatment groups (*n* = 6/group), which included three groups treated with Quercetin 10 mg/kg (1 mL/kg of a Quercetin 10 mg/mL solution in dimethyl sulfoxide 40%) [28, 29], followed 15 min later by water or NaNO_2_ 1 or 15 mg/kg by gavage [13, 30, 31], and three groups treated with vehicle (1 mL/kg of dimethyl sulfoxide 40%) followed 15 min later by water or NaNO_2_ 1 or 15 mg/kg by gavage. The doses of sodium nitrite were chosen with basis on previous studies showing that 1 mg/kg is a nonantihypertensive dose, whereas the 15 mg/kg exerts significant antihypertensive effects [32]. Drug treatments were carried out daily for 7 days. All drugs and reagents were purchased from Sigma Chemical Co. (St. Louis, MO, USA), and all solutions were freshly prepared just prior to use.

The number of animals per group proved to be sufficient to detect statistically significant biochemical differences when they existed in our previous studies. The experimental unit was the animal and we did not use a computer‐generated random sequence to allocate the animals to each study group. However, we found no significant differences between groups in their baseline characteristics. The researcher who performed the biochemical analyses was blinded to the experimental groups.

### 2.4 Evaluation of Systolic Blood Pressure

Systolic blood pressure (SBP) was assessed using the tail plethysmography method. For this purpose, a cuff attached to a pressure transducer was placed around the tails of awake animals, which had been previously warmed in cabinets set to 37°C. Pressure variations were captured by a specific data acquisition program using a PowerLab 4/S analog‐to‐digital converter (AD Instruments Ltd., Castle Hill, Australia), and the results were represented as the average of four measurements for each animal assessed on the seventh day of treatment.

### 2.5 Tissue Collection

On the seventh day, 40 min after drug treatment, the animals were anaesthetised with ketamine (100 mg/kg, i.p.) and xylazine (10 mg/kg, i.p.). The blood was collected directly from the cava vein into heparin‐containing tubes, followed by centrifugation to separate the plasma. The plasma was stored in tubes containing N‐ethylmaleimide (NEM) (10 mM) and diethylenetriaminepentaacetic acid (DTPA) (2 mM) to preserve nitrosylated species (RxNO) [33]. After blood collection, a catheter was inserted into the left ventricle, and the animals were perfused with a PBS solution at pH 7.4, which also contained NEM (10 mM) and DTPA (2 mM) [30]. Subsequently, the thoracic aorta was collected, immediately frozen in liquid nitrogen and stored at −80°C.

### 2.6 Measurement of Gastric NO Formation From Nitrite

Nitrite generates NO when exposed to the acidic environment of the stomach [14]. Therefore, we examined whether treatment with Quercetin enhances NO formation stimulated by treatment with oral nitrite, as detailed above. To measure gastric NO formation, the stomach was occluded at both ends (cardia and pylorus), and 5 mL of oxygen‐free nitrogen (N₂) were injected into the stomach. After 20 s, the gastric gas was collected and injected into the purge vessel connected to a NO analyser (Ecomedics Analyzer, CLD 88 sp.; Dürnten, Switzerland) to measure NO formation using chemiluminescence in the gas phase [14].

### 2.7 Determination of Nitrate, Nitrite and Nitrosylated Species Concentrations in Plasma and in the Aorta

Plasma samples were injected in natura, whereas aorta samples were homogenized using a glass homogenizer and phosphate‐buffered saline at pH 7.4, with added N‐ethylmaleimide 10 mM and diethylenetriaminepentaacetic acid 2 mM, which were added in order to preserve nitrosylated species [33]. For NO_2_^−^ measurement, plasma samples (50 μL) and aorta homogenates (30 μL) were injected into a solution of tri‐iodide and acetic acid in a system connected the NO chemiluminescence analyser, as previously described previously [34]. For NO_3_^−^ analysis, 20 μL of each sample were injected into a solution of vanadium chloride (VCl₂) in 1 M HCl at 96°C [30]. NO_3_^−^ and NO_2_^−^ concentrations in the aorta were normalized by the weight of tissue.

The determination of RxNO concentration was performed as previously described [35]. The samples (300 μL) were incubated with acid sulfanilamide (5% in hydrochloric acid) at a 10% volume ratio to the sample. After 3 min, the samples were injected into the tri‐iodide and acetic acid solution purged with nitrogen in line with the NO analyser.

### 2.8 Assessment of Protein Nitrosylation by Resin‐Assisted Capture Assay

To further explore the possibility that drug treatment could result in increased nitrosylation of tissue or plasma proteins, we used the S‐nitrosothiol resin‐assisted capture (SNO‐RAC) assay, as previously detailed [25, 26]. Aortic tissue proteins were extracted using lysis buffer (25 mM HEPES, 50 mM NaCl, 0.1 mM EDTA, 1% NP‐40, 0.1% SDS pH 7.4) supplemented with a protease inhibitor cocktail (Sigmafast, Sigma‐Aldrich). Homogenized samples were centrifuged at 12000 × *g* for 10 min at 4°C, and the supernatant was collected. Plasma samples were used in natura [36].

Each supernatant or plasma sample was incubated with 1.6 mL of blocking buffer (HEN buffer: 100 mM HEPES, 1 mM EDTA, 0.1 mM neocuproine; pH 8.1) plus 2.5% SDS and 20 mM methyl methanethiosulfonate. The mixture was incubated at 50°C for 20 min with gentle mixing every 5 min to block free thiol groups. Proteins were then precipitated by adding 6 mL of prechilled acetone and incubating at −20°C for 20 min. Samples were centrifuged at 2000 × *g* for 10 min at 4°C. The pellets were washed four times with 70% acetone and resuspended in 0.5 mL of HEN buffer containing 1% SDS. Samples were incubated overnight at 4°C in the dark with 20 mM ascorbate and 40 μL of thiopropyl‐sepharose 6B resin under gentle rotation. The resin was then washed four times with 1 mL of HEN buffer containing 1% SDS, followed by five washes with 1:10 diluted HEN buffer (HEN/10 + 1% SDS). Bound proteins were eluted by incubating the resin with HEN/10 buffer supplemented with 2% 2‐mercaptoethanol for 1 h at room temperature [37]. To quantify the percentage of nitrosylated proteins, the input (i) and output (o) samples were analysed on a 5% SDS‐PAGE gel combined with a 10%–5% gradient separating gel. Electrophoresis was halted once the protein front reached the 10% portion of the gel. The gels were stained with 0.05% Coomassie Brilliant Blue, and the band intensities were quantified using ImageJ software (NIH, USA).

### 2.9 Assessment of Vascular Reactive Oxygen Species Formation

#### 2.9.1 In Situ Detection of ROS Production

Vascular oxidative stress was assessed by reactive oxygen species (ROS) production in aortic tissue using dihydroethidium (DHE), a ROS‐sensitive fluorescent probe, as previously described [38]. Aortic cryosections (5 μm thick) were incubated with DHE (10 μmol/L) at room temperature for 30 min in a light‐protected, humidified chamber to prevent photooxidation. After incubation, the sections were washed with phosphate‐buffered saline and visualized using a fluorescence microscope (Leica Imaging Systems Ltd., Cambridge, UK). Images were captured at 400× magnification, and red fluorescence, indicative of ROS production, was quantified from 20 fields around the vessel using ImageJ software [39].

#### 2.9.2 Quantification of Lipid Peroxidation in the Plasma and in the Aorta

To assess lipid peroxidation in the plasma and in the aortic tissue, we measured thiobarbituric acid reactive substances (TBARS) using a fluorometric method, as previously described [40]. This method quantifies lipoperoxides expressed in terms of malondialdehyde concentrations using 1, 1, 3, 3‐tetramethoxypropane as standard. The method requires excitation at 515 nm and emission at 553 nm [40].

### 2.10 Statistical Analysis

Results were analysed using either unpaired Student's *t* test or two‐way analysis of variance (ANOVA), followed by Tukey's post hoc test for multiple comparisons. Differences were considered statistically significant at *p* < 0.05. Statistical analyses were performed using GraphPad Prism software, Version 8.0.1 (GraphPad Software Inc., San Diego, CA, USA).

## 3 Results

### 3.1 Quercetin Enhances NO Formation From NaNO_2_ and Potentiates Its Antihypertensive Effect

**FIGURE 1:**
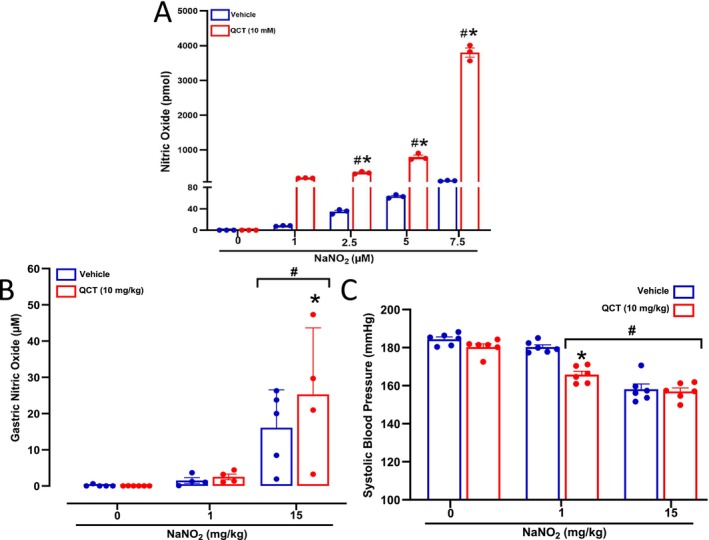
Quercetin enhances gastric formation of NO from nitrite and its antihypertensive effects. Panel A shows NO formation from NaNO_2_ at 1, 2.5, 5 and 7.5 μM concentrations added to a simulated gastric juice (SGJ) containing Quercetin (10 mM; red bars) or vehicle (blue bars). Panel B shows gastric NO formation assessed in rats treated with Quercetin 10 mg/kg (or vehicle) followed 15 min later by water or NaNO_2_ 1 or 15 mg/kg by gavage. After the stomach was occluded at the cardia and pylorus, 5 mL of oxygen‐free nitrogen (N₂) were injected into the stomach and the gastric gas was collected for NO concentration measurement using the NO analyser. Panel C shows the decreases in systolic blood pressure after water or NaNO_2_ 1 or 15 mg/kg by gavage in SHR previously treated with Quercetin 10 mg/kg (or vehicle). *p < 0.05 versus respective vehicle‐treated group. # *p* < 0.05 versus respective 0 dose or concentration.

To assess whether Quercetin enhances the reduction of NaNO_2_ to NO under acidic conditions, we employed a SGJ solution. Under these conditions, Quercetin (10 mM) significantly increased NO formation (> 3‐fold) from NaNO_2_ at 1‐, 2.5‐, 5‐ and 7.5‐μM concentrations (*p* < 0.05; Figure 1A). Next, we evaluated in vivo whether oral treatment with Quercetin would enhance in vivo gastric NO formation after oral nitrite administration. Figure 1B shows the concentrations of NO in the gastric gas collected from the stomach after NaNO_2_ 1 or 15 mg/kg administration in rats pretreated with Quercetin 10 mg/kg (or vehicle). While NaNO_2_ alone dose‐dependently increased NO concentrations, Quercetin enhanced NO formation from NaNO_2_ only at the 15 mg/kg dose (*p* < 0.05; Figure 1B). Oral nitrite treatment dose‐dependently decreased SBP (*p* < 0.05; Figure 1C), although the 1 mg/kg did not exert antihypertensive effect, as expected [32]. Interestingly, although Quercetin alone did not lower blood pressure, this drug converted a nonantihypertensive dose of nitrite (1 mg/kg) into an effective antihypertensive intervention (*p* < 0.05; Figure 1C), even though it did not enhance the responses to the high nitrite dose (15 mg/kg).

### 3.2 Quercetin Enhances the Antihypertensive Effects of NaNO_2_ Without Increasing Circulating or Aortic Nitrate and Nitrite Concentrations

Given that Quercetin enhanced the antihypertensive responses to nitrite treatment, we examined whether Quercetin treatment would result in higher nitrate or nitrite concentrations in plasma or in aortic samples that could helpexplain the blood pressure responses.

**FIGURE 2:**
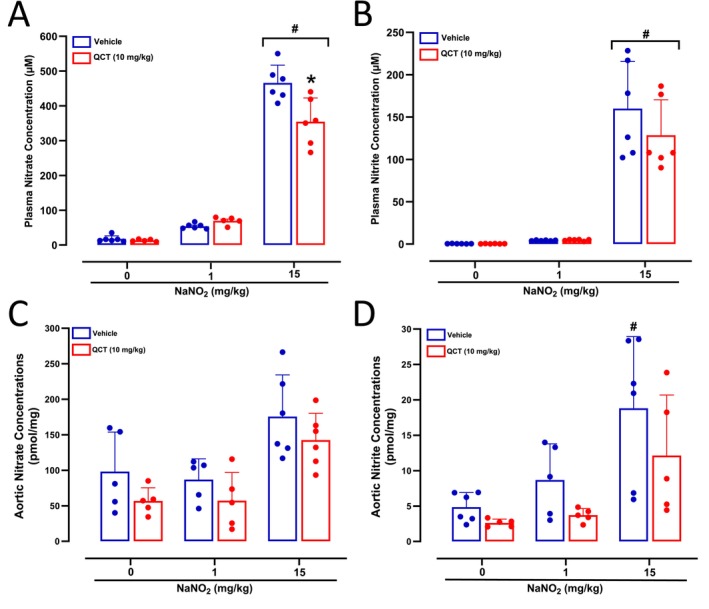
Plasma and aortic nitrate and nitrite concentrations after Quercetin and NaNO_2_ treatments. Panels A and B show plasma nitrate and nitrite concentrations, respectively, measured in plasma samples from SHR treated with Quercetin 10 mg/kg (or vehicle), followed by water or NaNO_2_ 1 or 15 mg/kg administered by gavage. Panels C and D show aortic nitrate and nitrite concentrations, respectively, measured in aortas from SHR treated with Quercetin 10 mg/kg (or vehicle), followed by water or NaNO_2_ 1 or 15 mg/kg administered by gavage. *p < 0.05 versus respective vehicle‐treated group. #*p* < 0.05 versus respective 0 dose or concentration.

While nitrite treatment at 15 mg/kg increased plasma nitrate concentrations (*p* < 0.05; Figure 2A), pretreatment with Quercetin attenuated this effect by < 20% (*p* < 0.05; Figure 2A). The circulating concentrations of nitrite increased only after nitrite administration at 15 mg/kg (*p* < 0.05; Figure 2B), without significant effects of Quercetin pretreatment.

The nitrate and nitrite aortic concentrations showed a similar behaviour. Only nitrite administration at 15 mg/kg tended to increase aortic nitrate concentrations (*p* < 0.10; Figure 2C), without significant effects of Quercetin pretreatment. Similarly, only nitrite administration at 15 mg/kg increased aortic nitrite concentrations (*p* < 0.05; Figure 2D), but not in SHR pretreated with Quercetin.

### 3.3 Quercetin Enhances the Antihypertensive Effect of NaNO_2_ Without Increasing Protein S‐Nitrosylation

**FIGURE 3:**
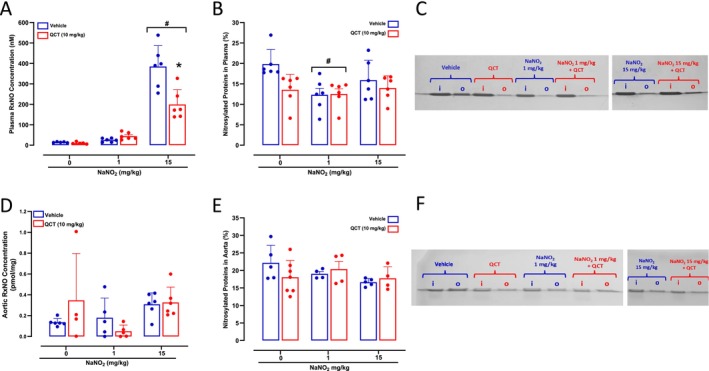
Plasma and aortic total protein nitrosylation after Quercetin and NaNO_2_ treatments. Panels A and B show plasma nitrosylated species (RxNO) concentrations assessed by chemiluminescence and the percentage of nitrosylated proteins assessed by the SNO‐RAC assay, respectively, in plasma samples from SHR treated with Quercetin 10 mg/kg (or vehicle), followed by water or NaNO_2_ 1 or 15 mg/kg administered by gavage. Panel C shows a representative SDS/PAGE gel stained with Coomassie Blue to quantify total protein nitrosylation in plasma samples from the SHR using the SNO‐RAC method (‘I’ corresponds to input: total protein; ‘o’ corresponds to output: nitrosylated protein). Panel D and E show aortic nitrosylated species (RxNO) concentrations assessed by chemiluminescence and the percentage of nitrosylated proteins assessed by the SNO‐RAC assay, respectively, in aortas from SHR treated with Quercetin 10 mg/kg (or vehicle), followed by water or NaNO_2_ 1 or 15 mg/kg administered by gavage. Panel F shows a representative SDS/PAGE gel stained with Coomassie Blue to quantify total protein nitrosylation in aortas from the SHR using the SNO‐RAC method. *p < 0.05 versus respective vehicle‐treated group. #*p* < 0.05 versus respective 0 dose or concentration.

While treatment of hypertensive rats with NaNO_2_ 1 mg/kg had no effects on plasma RxNO concentrations, the 15 mg/kg dose increased RxNO significantly, and Quercetin attenuated this effect (*p* < 0.05; Figure 3A). We found lower concentrations of plasma nitrosylated proteins after NaNO_2_ 1 mg/kg (*p* > 0.05; Figure 3B,C), whereas the 15 mg/kg and Quercetin treatments had no effects. Interestingly, these changes in plasma concentrations of NO metabolites after NaNO_2_ and Quercetin treatments were not associated with any significant changes in both RxNO and plasma nitrosylated proteins measured in the aorta (all *p* > 0.05; Figure 3D–F).

### 3.4 Quercetin and NaNO_2_ Reduce Oxidative Stress When Administered Alone or in Combination

**FIGURE 4:**
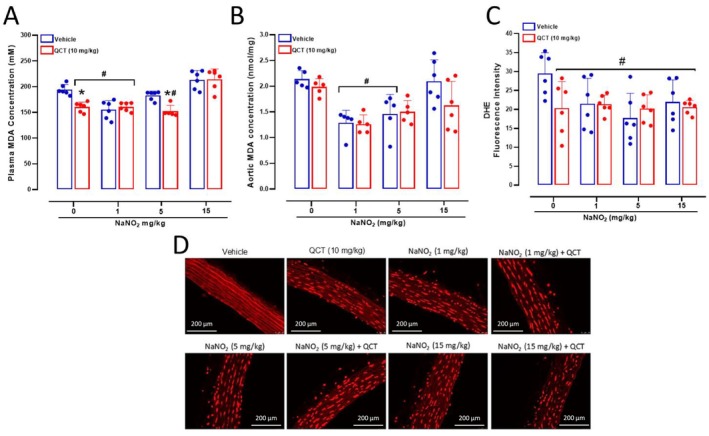
Antioxidant effects of Quercetin and nitrite treatments in SHR. Panel A and B shows plasma and aortic lipid peroxidation, respectively, measured as thiobarbituric acid reactive substances and expressed in terms of malondialdehyde (MDA) concentrations measured in samples from SHR treated with Quercetin 10 mg/kg (or vehicle), followed by water or NaNO_2_ 1 or 15 mg/kg administered by gavage. Panel C shows in situ aortic reactive oxygen species formation measured by dihydroethidium (DHE) fluorescence. Panel D shows representative photomicrographs (original magnification, 400×) of aortas incubated with DHE to produce red fluorescence. *p < 0.05 versus respective vehicle‐treated group. #*p* < 0.05 versus respective 0 dose or concentration.

We evaluated lipid peroxidation to assess oxidative stress both in plasma and in the aorta from SHR treated with Quercetin and different doses of NaNO_2_. The results show that Quercetin 10 mg/kg alone reduced plasma MDA levels (*p* < 0.05; Figure 4A), and NaNO_2_ 1 mg/kg alone or combined with Quercetin resulted in similar antioxidant effects (*p* < 0.05; Figure 4A). The assessment of MDA concentrations in the aorta, however, showed that Quercetin alone did not significantly affect aortic MDA concentrations (*p* > 0.05; Figure 4B) and that treatment with NaNO_2_ 1 mg/kg, either alone or combined with Quercetin, reduced MDA concentrations as compared to the vehicle group (*p* < 0.05; Figure 4B).

To further explore antioxidant responses to either Quercetin or NaNO_2_, we also assessed the formation of reactive oxygen species in the aorta from SHR. The results of DHE assay showed that Quercetin alone or NaNO_2_ at 1 or 15 mg/kg resulted in antioxidant effects, as revealed by DHE fluorescence signal (all *p* < 0.05; Figure 4C,D). However, no significant interaction was found between Quercetin and NaNO_2_ treatments (*p* > 0.05; Figure 4C,D).

## 4 Discussion

The findings of this study provide novel evidence that Quercetin treatment converted a nonantihypertensive dose of nitrite into an effective antihypertensive intervention in association with increased gastric NO formation, without further increasing nitrite‐induced increases in systemic or vascular nitrite/nitrate levels, or vascular protein nitrosation. These findings suggest a potential therapeutic strategy to improve the efficacy of dietary or pharmacological nitrite by leveraging the redox properties of Quercetin.

We first provided in vitro evidence that Quercetin markedly enhanced NO formation from nitrite in SGJ under acidic conditions, consistent with its previously reported nitrite‐reducing capacity [21, 41]. Importantly, this chemical facilitation was also confirmed in vivo, and Quercetin pretreatment increased gastric NO release after oral nitrite treatment. These results support the hypothesis that Quercetin can act as a gastric NO enhancer and possibly potentiate the functional consequences of increased gastric NO formation after oral nitrite administration [10].

The antihypertensive properties of orally administered NaNO_2_ reported here have been consistently shown in different hypertension models [42, 43], including the L‐NAME [44], the DOCA‐salt [45] and the two‐kidney, one‐clip [32] models. Supporting our findings, previous studies have shown that NaNO_2_ significantly reduces blood pressure in a dose‐dependent manner by mechanisms that result in increased NO activity [12, 45, 46] or increased endothelium‐dependent NO formation [47]. However, taking into consideration that hypertension is a chronic condition requiring long term therapy and that nitrite may offer some toxicity including potential adverse effects associated with prolonged exposure, it would be very helpful to define strategies that minimize the doses of nitrite producing relevant effects. To achieve this goal, combining nitrite with other antioxidant drugs, such as ascorbic acid, which may potentiate its effects and reduce the formation of nitrosamines has been suggested [17, 31, 48]. Here, we report for the first time that Quercetin converted a nonantihypertensive dose of nitrite into an effective antihypertensive dose, thus suggesting that Quercetin, a well‐recognized antioxidant drug, may help reduce nitrite to NO [21]. In fact, we found that Quercetin combined with nitrite at 1 mg/kg resulted in blood pressure responses similar to those found with nitrite 15 mg/kg alone (Figure 1C). These responses are comparable with those previously reported when nitrite was administered combined with tempol [31] or ascorbate [17] and suggest that Quercetin enhances the blood pressure responses to oral nitrite administration, thus minimizing the doses of nitrite required to decrease the blood pressure. This is a very convenient finding because it supports the idea that nitrite may be used at a low dose when combined with drugs that may enhance its NO‐generating properties.

While Quercetin functionally potentiated the antihypertensive effects of nitrite at low dose in association with increased gastric NO formation, this effect was selective to the gastric compartment, as systemic concentrations of nitrite, nitrate and RxNOs were not increased by Quercetin co‐treatment. Therefore, this functional effect was not attributable to increased systemic bioavailability of nitrite, nitrate or other nitrosylated species, as previously reported with oral nitrite treatment [9, 25, 26]. While our findings do not support a role for enhanced vascular protein S‐nitrosation in mediating these responses, it is possible that increased local gastric NO production might be sufficient to exert systemic vasodilatory effects, although the mechanisms explaining such an effect are not clear at this time and require further investigation. In addition, nitrite supplementation has shown antihypertensive effects associated with improved vascular function and antioxidant responses in a variety of previous studies [46, 49, 50], and those effects are similar to those found with the flavanoid Quercetin, which also produces antioxidant and antihypertensive effects [18, 19, 20, 21, 22, 23, 24]. Although unproven, it is probable that both treatments with Quercetin and nitrite interacted in the present study to result in improved antihypertensive responses by increasing NO activity in the vasculature as a result of their antioxidant effects, even though we found no additive antioxidant effect in the present study. This suggestion is based on the overall trend for increased nitrite or nitrate concentrations in the aortas after both treatments, even though those effects were not consistently significant.

Interestingly, Quercetin pretreatment attenuated nitrite‐induced rise in plasma RxNO concentrations, suggesting that it may buffer nitrite‐induced formation of nitrosylated species, which depend on low gastric pH conditions [11, 12, 13, 14]. This is an unexpected finding in the context of Quercetin‐induced increase in NO formation from nitrite in SGJ, both in vitro and in vivo, as reported here. Indeed, nitrite is protonated in the acidic environment of the stomach and forms nitrous acid, which undergoes other chemical reactions producing dinitrogen trioxide and other nitrosylated species [12]. While these chemical reactions should be enhanced by Quercetin [21, 41], it should be also taken into consideration that both the stomach and the intestine express xanthine oxidoreductase (XOR), which plays a critical role in the formation of NO and other nitrosylated species after oral nitrite administration [51]. Given that Quercetin inhibits XOR [52, 53], it is highly probable that Quercetin may have simultaneously facilitated NO formation from nitrite when both drugs interacted in the gastric juice [21] and inhibited NO formation derived from XOR‐mediated nitrite reductase activity [51], thus resulting in lower final NO yields after nitrite administration, as suggested by our results. This dual interaction may have blunted the formation of RxNO and the accumulation of NO metabolites in the tissues after oral nitrite treatment [54].

This study has some limitations. First, Quercetin enhanced gastric NO formation following oral administration of NaNO_2_, and this effect was observed only with the high dose of NaNO_2_. However, part of NO generated after nitrite administration may have been eliminated by belching and therefore not taken into account, potentially leading to an underestimation of the measured values. Second, we have not defined a mechanism that could explain how Quercetin pretreatment increased gastric NO release after oral nitrite treatment and enhanced it antihypertensive effect. However, we believe that this is an important observation that will require further mechanistic studies. Third, the SHR animal model used here may be limited when extrapolating our findings to humans and requires caution, especially given species‐specific differences in nitrite metabolism and gastric physiology. Finally, there was substantial variation in the RxNO measurements reported in this study, particularly when assessed in aortic samples, even though this method has a 3.8% the coefficient of variation in our hands. We used the Grubbs' test to determine whether the most extreme value in the dataset was a significant outlier and we found that none of the values differed significantly from the others (*p* > 0.05). However, it should be noted that we confirmed our finding using the SNO‐RAC method.

In conclusion, our study shows that Quercetin is a potent enhancer of gastric NO formation from nitrite and is capable of amplifying its antihypertensive effects. These findings suggest that combining supplementation of flavonoids with nitrite‐based therapies may represent a safe and effective strategy in the therapy of cardiovascular diseases.

## Funding

This work was supported by Fundação de Amparo à Pesquisa do Estado de São Paulo (2023/07565‐5), Conselho Nacional de Desenvolvimento Científico e Tecnológico (406442/2022‐3) and Coordenação de Aperfeiçoamento de Pessoal de Nível Superior (Finance Code 001).

## Conflicts of Interest

The authors declare no conflicts of interest.

## Data Availability

The data that support the findings of this study are available from the corresponding author upon reasonable request.
